# Effects of Tumor-Derived DNA on CXCL12-CXCR4 and CCL21-CCR7 Axes of Hepatocellular Carcinoma Cells and the Regulation of Sinomenine Hydrochloride

**DOI:** 10.3389/fonc.2022.901705

**Published:** 2022-07-04

**Authors:** Conghuan Shen, Jianhua Li, Ruidong Li, Zhenyu Ma, Yifeng Tao, Quanbao Zhang, Zhengxin Wang

**Affiliations:** General Surgery Department, Affiliated Huashan Hospital of Fudan University, Shanghai, China

**Keywords:** hepatic carcinoma, CXCL12-CXCR4, CCL21-CCR7, sinomenine hydrochloride, tumor-derived DNA

## Abstract

Currently, chemokines and their receptors, CXCL12-CXCR4 and CCL21-CCR7 axes, are deemed vital factors in the modulation of angiogenesis and are crucial for the growth and development of liver cancer. Tumor-derived DNA can be recognized by immune cells to induce an autoimmune response. In this study, we demonstrated the mechanism of tumor-derived DNA on the CXCL12-CXCR4 and CCL21-CCR7 axes of hepatocellular carcinoma (HCC) cells and the regulatory effect of sinomenine hydrochloride. Tumor-derived DNA was separated from HCCLM cell lines. Tumor-derived DNA was transfected into SK-Hep1 cells by Lipofectamine 2000. We found that sinomenine hydrochloride reduced the expression of CXCR4, CXCR12, CCR7, and CCL21 in HCC cells, suppressed the growth and invasion of HCC cells, and increased apoptosis. In contrast to the controls, the protein expressions of CXCR4, CXCL12, CCR7, CCL21, P-ERK1/2, MMP-9, and MMP-2 in SK-Hep1 cells were significantly increased after transfection of tumor-derived DNA, while the increase was reversed by sinobine hydrochloride. Acid sinomenine interferes with tumor-derived DNA and affects ERK/MMP signaling *via* the CXCL12/CXCR4 axis in HCC cells. CXCR4 siRNA and CCR7 siRNA attenuated tumor-derived DNA activation of ERK1/2/MMP2/9 signaling pathways in HCC cells. CXCR4-oe and CCR7-OE enhance the stimulation of erK1/2/MMP2/9 signaling pathway by tumor-derived DNA in HCC cells. Tumor-derived DNA reduced apoptosis and increased invasion of SK-Hep1 cells by CXCL12-CXCR4 axis and CCL21-CCR7 axis, and sinobine hydrochloride reversed this regulation. These results strongly suggest that tumor-derived DNA can increase the growth and invasion of oncocytes *via* the upregulation of the expression of CXCL12-CXCR4 and CCL21-CCR7 axis and through ERK1/2/MMP2/9 signaling pathway in HCC cells, and sinobine hydrochloride can inhibit this signaling pathway, thus inhibiting HCC cells. These results provide new potential therapeutic targets for blocking the progression of HCC induced by CXCL12-CXCR4 axis and CCL21-CCR7.

## Introduction

Hepatic carcinoma is one of the most commonly seen malignancies of the digestive system in the world. In 2020, there were 905,677 novel cases of liver carcinoma across the globe, accounting for 4.7% of the novel tumor cases in the whole year, making it the second leading cause of tumor death in the world, with 830,180 deaths (8.3%) in the whole year (1, 2). Hepatocellular carcinoma (HCC), the most commonly seen type of primary hepatic carcinoma, is one of the most commonly seen malignancies in clinical practice, and its related mortality ranks the third among malignant tumors (1). Most patients with HCC have no obvious symptoms at the early stage but are already in the middle and advanced stages when they are treated, and the overall prognosis is still poor (3). It was found that the great rate of cancer relapse and metastases was the main factor leading to unsatisfactory prognoses of HCC sufferers. Hence, new enlightenment pertaining to the mechanisms of HCC is imperative for the determination of new prognosis molecule biomarkers and potentially valid treatment targets to ameliorate the survival of sufferers (4, 5).

When tumor cells are necrotic, normally apoptotic, and killed by chemotherapy and radiotherapy, a large amount of DNA can be repositioned from the nucleus and released into the circulatory system. The released DNA can lead to autoimmune reactions, and the circulating DNA can stimulate immunocytes, like macrophages and natural killer cells, and then induce autoimmune reactions (5, 6). Circulating tumor DNA (ctDNA) is a specific tumor-related DNA fragment that is secreted and released into peripheral blood during tumor cell renewal, necrosis, and apoptosis. ctDNA can reflect tumor cytogenetics and epigenetic information (7). In the whole-cycle management of HCC, ctDNA monitoring can dynamically provide the information of tumor genetic variation, which has become a hot research direction in recent years (8).

Sinomenine is the alkaloid monomer extracted from Chinese traditional medicine. Sinomenine hydrochloride (SIN) is a water-soluble hydrochloride, which has anti-inflammatory and anti-immune pharmacological effects. Recent studies have also found that sinomenine can inhibit the proliferation, block the cell cycle and induce the apoptosis of oncocytes such as hepatic carcinoma, gastric cancer, mammary carcinoma, and osteosarcoma. Li et al. displayed that SIN can reduce breast cancer metastasis by inducing breast cancer cell death and inhibiting inflammatory associated epithelial mesenchymal transition (9). Deng et al. discovered that SIN promotes programmed cell death in kidney cell cancer by increasing autophagic activities *via* the phosphatidylinositol 3-kinase/AKT/rapamycin path (10). In addition, research has revealed the integrated effects of SIN and chemotherapeutic drugs in cancer treatment. Liu et al. found that SIN enhanced doxorubicin sensitivity of colon cancer cells (CACO-2) by downregulating the expression of multiple drug-tolerant protein 1 and cyclooxygenase-2 (11). In addition, the integrated effect of SIN and 5-fluorouracil on esophageal cancer is superior to that of 5-fluorouracil alone and does not aggravate the adverse events of chemotherapies. However, the potential mechanism of SIN in inhibiting HCC cells remains to be fully elucidated.

This study will investigate the effects of CXCL12-CXCR4 and CCL21-CCR7 axis signaling pathways on cancer-derived DNA activation and proliferation induction of HCC cells. Experiments were conducted to elucidate that SIN inhibits tumor-derived DNA tumor-induced HCC cells and induces cell proliferation through CXCL12-CXCR4 and CCL21-CCR7 axis signaling pathways, aiming to provide possible drug selection and relevant preclinical evidence for clinical HCC treatment.

## Methods

### Reagents and Antibodies

SIN was bought from Zheng Qing Pharmaceutic Group (Hunan, PRC). Human HCC cell line SK-Hep1 (Shanghai Aolu Biotechnology Co., Ltd.), TRIzol reagent, SuperScript III first-strand synthetic system (Invitrogen, America), ABI 7500 fast real-time PCR system (Applied Biosystems, USA), diquinone bicinchoninic acid (BCA) tool (Thermo Fisher Scientific, USA), Fluorchem FC3 system (Proteinsimple, USA), polyvinylidene fluoride (PVDF) membrane, chemiluminescent reagent (Millipore, USA), transpore Chamber (BD Biosciences, USA), diaminophenylindole (DAPI), 3-MA, 4-PBA, LPS, and MDC were bought from Sigma-Aldrich (USA), Cell Counting Kit 8 (CCK-8; Japan Dojindo Company), multi-hole scanning spectrophotometer (US Thermo Scientific Company). Single-person ultra-clean bench (Beijing Liuyi Instrument Factory), CB15 C02 cell incubator (Beijing Liuyi Instrument Factory), Victor3 1420 Multi-lable Counter microplate reading device (BD, USA), HD-3000 gel imaging device (Shang Tian Precision Apparatus Company, PRC), Millipore flow cytometer (Beijing Weixin Yiao Technology Development Co., Ltd.). Phospho-ERK1/2, matrix metalloproteinase (MMP)-2, MMP-9, extracellular signal-regulated kinase 1/2 (ERK1/2), CXCL12, CXCR4, CCL21, CCR7, Caspase 3, and GAPDH antibodies were bought from Abcam (UK). Every second antibody was provided by Santa Cruz Biotechnology, Inc. (USA).

### Cell Culture

Human HCC cell line SK-Hep1 was cultivated in dulbecco's modified eagle medium (DMEM) or Roswell Park Memorial Institute (RPMI) 1640 intermediary with 10% FBS. The medium involved 100 mg/ml penicillin and kyowamycin sulfate. And in 5% CO_2_, 90% humidity, 37°C incubator. Harvested media and cell lysates are preserved under -20°C for subsequent assays. Mouse liver cancer cell lines (Hepa1-6) were cultivated in DMEM Basic medium, 10% FBS, 100 U/ml penicillin-kyowamycin, 37°C, 5% CO_2_ cell box. Cellular cultivation intermediary was refreshed every 1–2 days, and cell passage was performed when 90% of cells were fused.

### Tumor-Derived DNA Transfection

Serum-free medium was mixed with Lipofectamine 2000 5 μl every 50 μl and let stand for 5 min. Serum-free medium was mixed with extracted tumor cell (Lewis cell) DNA every 50 μl and let stand for 5 min. The two were fully mixed and let stand for 20 min. The transfected cells were cleaned two times in serum-free DMEM/F12, and the mixed solution of liposome and DNA was supplemented to the cells. Cells were cultivated under 37°C and 5% CO_2_ for 4–6 h. The transfection intermediary was removed, and 10% FBS-DMEM/F12 intermediary was supplemented into the cells, and the cells were cultured for 48 h. Real-time PCR was leveraged to identify the expression of 18S mRNA to determine the transfection effect, and then Western blot (WB) or other experiments were performed.

### Transfection of siRNA

SiRNA-CXCR4 plasmid, siRNA-CCR7 plasmid, and negative control plasmid (siRNA-NC) were all provided by Genechem Shanghai, Primer sequences: GATGAGGTCACGGACGATT (5’-GATCCGATGAGGTCACGGACGATTCTATGGACAAATCGTCCGTGACCTCATCTTTTTTGTCGACA-3’, 3’-GCTACTCCAGTGCCTGCTAAGATACCTGTTTAGCAGGCACTGGAGTAGAAAAAACAGCTGTTCGA-5’). The transfection was performed according to the Lipofectamine™ 2000 (Invitrogen) kit instruction. Trypsinase was digested before transfection, and the cell density was modified to 6 × 10^6^/ml in RPMI 1640 intermediary involving 10% FBS. Cells were inoculated in 24-well culture dishes nightlong and transfected when the cell culture reached about 75% after fusion. At 48 h after transient transfection, the digested cells were inoculated on 6-well dishes and cultivated with RPMI 1640 intermediary for subsequent assay.

### Transfection of Plasmid DNA (CXCR4/CCR7 High-Expression Plasmid Screening Validation)

The cell culture medium in the 6-well plate was changed to 1.5 ml DMEM(-), and 12 1.5-ml EP tubes were placed on the test tube shelf. The cells were equally divided into two groups: Lipofectamine^®^2000 Transfection Reagent group and plasmid DNA group. In this study, 245, 245, 245, 240, 240, and 240 μl DMEM(-) were added to each tube of the Lipofectamine^®^2000 Transfection Reagent. The required DMEM(-) volume was calculated according to the measured plasmid concentration in the plasmid DNA set. Add 5μL, 5μL, 5μL, 8μL, 8μL, 8μL Lipofectamine®2000 Transfection Reagent to the Lipofectamine®2000 Transfection Reagent group, and mix well. 2, 4, and 6 μg pcmv3-CXCR4-ha plasmids were added to the plasmid DNA groups and mixed and stood at room temperature for 5 min. Subsequently, the plasmid DNA group and the Lipofectamine^®^2000 Transfection Reagent group were mixed in pairs and stood at room temperature for 20 min to form the composite. The mixture was evenly dropped into the corresponding culture well, and the compound was evenly distributed in the medium by gently shaking the 6-well plate, and the label was made. At 4–6 h later, 200 μl FBS was added to each well. Cells were observed the next day and collected 48 h later.

### Total RNA Extraction and Fluorescence Quantitative PCR

After trypsin digested cells in each experimental group, cells were lysed with TRIzol solution, total RNA was abstracted as per the specification of RNA tool, and RNA was reversely transcribed to cDNA as per the specification of TaqMan reverse transcription kit. According to the instructions of Takara fluorescence quantitative kit, RT-qPCR was performed on Light Cycler480II fluorescence quantitative PCR instrument. GAPDH was utilized as an internal reference, and 2^-ΔΔCt^ was employed to compute the relative expression levels (12). All experiments were repeated in triplicate. Primer sequences are as follows:

Mmp-9, 5’-ATGGAGCTGGAATTGGATGC-3’ (forward), 5’ -cTAGCCATATCTGTCCT-3 ‘ (reverse); Mmp-2, 5’-tacacctataccaagaACTTCCG-3’ (forward), 5’-tgTCCGCCAGatGaACCG-3’ (reverse); CCR7, 5’-AAGGGTCAGGAGGAAGagGA3’ (forward), 5’-GGCTGGTCGTGTTGACCtAT-3’ (reverse); GAPDH, 5’-TCACTGCCaagaaga-3’ (forward), 5’-TaccagGAaatGAGcTTGA-3’ (reverse). Real-time fluorescence quantitative PCR was completed using the QuantiTect SYBR Green RT-PCR tool (QIAGEN, Germany) *via* ABI Prism 7000 sequence detecting device (Applied Biological Systems, Germany). The comparative level of MMP-2/-9 mRNA was normalized to GAPDH mRNA. The assay was carried out in triplicate.

### Detection of Cell Proliferation Activity

The proliferative activity of cells was identified *via* CCK-8 approach. The transfected cells were digested with trypsinase, and the cellular density was modified by medium. Here, 10^4^ cells in each well were seeded to 96-well plates at 0, 24, 48, and 72 h, 10 μl CCK-8 liquor was supplemented, mixed and incubated in an incubator for 4 h. Optical density (OD) was identified at 490 nm *via* an automatic porous spectral photometer (Bio-Rad Laboratories, USA). Cellular proliferation curves were plotted using culture time as abscissa and OD value as ordinate.

### MTT Cell Proliferation Assay

Single-cell suspension was prepared from P3 Bone Mesenchymal Stem Cells (BMSCs), the cellular density was modified (5 × 10^4^ cells/ml), and cells were seeded to a 96-well dish (5 × 10^3^ cells/well). Following cell adherence, the cultivation intermediary was cultured for 24 h, and amaranth was supplemented into the cultivation intermediary at a content gradient of 5, 10, and 20 μg/ml for 48 h. 3-(4,5)-dimethylthiahiazo (-z-y1)-3,5-di- phenytetrazoliumromide (MTT) 20 μl (5 mg/ml) was added, culturing in 37°C incubator was continued for 4 h, the supernatant was absorbed, DMSO was added to each well, 150 μl was vibrated for 10 min, and the absorbance value (OD490 nm) was measured with a microplate reader.

### Transwell Experiment

After serum-free culture for 12 h before inoculation, P3 cells were digested and the cellular concentration was modified to 2 × 10^5^ cells/ml in FBS medium (0.1%). In this study, 200 μl cellular suspension was supplemented into the upper chamber. In addition, 750 μl FBS intermediary (20%) with or without amaranth (5, 10, 20 μg/ml) was supplemented into the lower chamber, and 3 multiple wells were set for every group. After 10-h culture in the incubator, the chamber was taken out, the cultivation intermediary in the abandoned well was cleaned two times in PBS, subjected to fixation in 4% PFA for 20 min, cleaned two times in PBS, dyed with Giemsa dye for 20 min, and cleaned two times in PBS. Under an inverted microscope (×400), 5 fields were randomly selected for observation of counting cells.

### Detection of Apoptosis

Annexin V and propidium iodide (PI) apoptotic identification tool was leveraged to identify programmed cell death. Cells in each transfection group were subjected to digestion *via* trypsin, and the cellular concentration was modified to 1 × 10^6^/ml by resuspension combined with buffer. Annexin V 5 μl and PI 5 μl were supplemented into suspension cells and mixed. The cells should be protected from light for 5–15 min under ambient temperature. After the cellular suspension was mixed, it was identified *via* flow cell technique immediately. The excitation wavelength Ex = 488 nm and emission wavelength Em = 530 nm and the cell suspension could be lightly elastic again to avoid cell clumps before the machine.

### Western Blotting Experiment

The cells were cultured in a 10-cm Petri dish, and after the degree of cell fusion reached over 90%, cell precipitation was collected, and RIPA protein lysate containing Phenylmethanesulfonyl fluoride (PMSF) was added to fully lysate the cells. After the content of every specimen was identified *via* BCA protein quantitative kit, the samples were stored in a refrigerator at -80°C for use. The WB procedure was as follows: 50 μg total protein was electrophoresized in 12% sodium dodecyl sulfate (SDS)-polyacrylamide gel (110 V). The proteins in the gel were moved onto the PVDF film (current: 300 mA, 2 h). Here, 5% powdered skimmed milk was sealed under ambient temperature for 3 h. Tween-20 phosphate buffer (PBST) was washed 3× 5 min, and primary antibody was incubated at 4°C overnight (β-actin, CXCL12, CXCR4, CCL21, CCR7, lysed Caspase 3, caspase 3, The concentrations of ERK and P-ErK were 1:1,000), and the PBST membrane was washed for 3× 5 min. Horseradish peroxidase (HRP)-labeled second antibody (1:10,000) was cultivated under 37°C for 45 min. PBST washed the membrane 4× 5 min. Electrochemiluminescence (ECL) developer was supplemented to the membrane, and the membrane was exposed and photographed in a fully automatic chemiluminescence system.

### Immunofluorescence

After digestion and centrifugation, the cells were seeded to a 6-well dish with cover glass at a rate of 5 × 10^5^/well, and 2 ml/well of DMEM/F12 medium with 10%FBS was added and placed in an incubator overnight. DMEM/F12 was cleaned twice, SIN was added with a concentration of 0.25 mM, and cultured for 48 h. Lipopolysaccharide (LPS) 2 ml with the concentration of 1 μg/ml was added and cultured for 4 h. The intermediary was discarded and cleaned in PBS 3 times. Here, 2 ml of 4% paraformaldehyde was added into the well and fixed overnight at 4°C. Then, 4% paraformaldehyde was discarded and cleaned with PBS 3 times. Then, 0.1% Triton-100 was added, 2 ml/well, 10 min later, it was washed with PBS 3 times. The primary antibody was added at 4°C overnight, negative control PBS 30 μl, and washed with PBS 3 times. After adding second antibody for 1 h, it was washed with PBS 3 times. DAPI 30 μl/tablet (diluted at 1:2,000) was added, washed with PBS 3 times under ambient temperature for 5 min. Immunofluorescence blocker was used to block the cells, and the cells were analyzed and imaged under a fluorescent microscope.

### Wound Healing Test

The cells were digested with trypsin and inoculated evenly into a six-well tissue culture plate 1 day before the scratch and grew to almost complete confluence within 24 h. A sterile 10-μl tip was used to generate an artificial homogeneous wound on a single layer. After scraping, the cells were cleaned in serum-free intermediary. Pictures of cell migration to the wound were collected at 0 and 24 h, and every assay was completed ≥3 times.

### Statistics

SPSS 11.0 statistic program package was utilized for statistical assays, and the data were presented as average ± SD (X ± S). The mean of multiple samples was studied *via* one-way ANOVA. Least—Significant Difference (LSD) method was utilized to contrast the experiment group with the controls. Snk-q test was employed to contrast the mean number of several samples. P < 0.05 had significance on statistics. GraphPad software was used to draw relevant figures (Insightful Science company).

## Results

### CXCL12-CXCR4 Axis Participated in the Effect of Sinomenine Hydrochloride on Hepatocellular Carcinoma Cells

**Figure 1:**
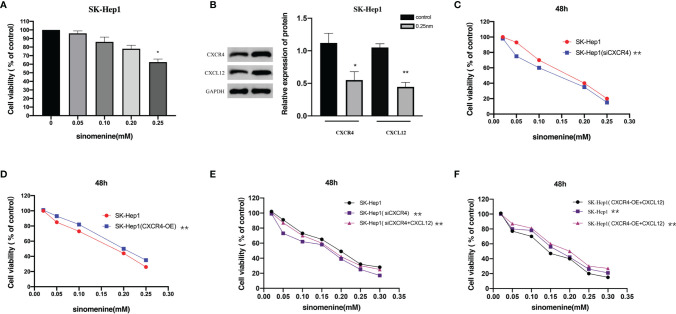
The CXCL12-CXCR4 axis is involved in the role of sinomenine hydrochloride in the proliferative activity of hepatocellular carcinoma (HCC) cells. **(A)** 3-(4,5)-dimethylthiahiazo (-z-y1)-3,5-di- phenytetrazoliumromide (MTT) assay to assess the viability of SK-Hep1 cells exposed to diverse levels of sinomenine hydrochloride. **(B)** Western blot (WB) to detect the expression of CXCL12 and CXCR4 in SK-Hep1 cells. **(C)** Decrease of CXCR4 expression by siCXCR4 in SK-Hep1 cells, SK-Hep1 cellular activity was identified *via* CCK-8 analysis. **(D)** SK-Hep1 cellular activity was identified in SK-Hep1 cells by overexpressing CXCR4, Cell Counting Kit-8 (CCK-8) assay. **(E, F)** CCK-8 assay measures cell viability. *P < 0.05, **P<0.01.

Firstly, we detected the effect of SIN on HCC cell viability. SK-Hep1 cells were exposed to SIN at different levels for 48 h, and cellular activity was evaluated *via* MTT assay. **Figure 1A** shows the roles of SIN in the viability of human liver cancer SK-Hep1 cells. The outcomes revealed that the cell viability of SK-Hep1 was significantly reduced by 0.25-mM sinoline hydrochloride for 48 h. CXCL12/CXCR4 axis is widely believed to be vital for many tumors and metastases, including liver carcinoma (13). To determine how SIN affects the proliferative and invasive activities of HCC cells, we subsequently investigated whether SIN regulates the expression of CXCL12/CXCR4 in HCC. As shown in immunoblotting, the expressions of CXCL12 and CXCR4 were remarkably downregulated in SK-Hep1 cells 48 h after 0.25-mM SIN treatment, suggesting that the CXCL12-CXCR4 axis was affected by sinine in HCC cells (**Figure 1B**). Subsequently, we significantly reduced CXCR4 expression by siCXCR4 in SK-Hep1cells, and then SK-Hep1 cells were treated with diverse levels of sinocine hydrochloride for 48 h. Cellular activity was identified *via* CCK-8 analysis. The outcomes revealed that the cell viability of SK-Hep1 cells with CXCR4 knockdown was significantly reduced by comparison (**Figure 1C**). On the contrary, the activity of SK-Hep1 cells overexpressing CXCR4 was remarkably increased (**Figure 1D**). In addition, SK-Hep1 cells transfected with CXCR4 presented a remarkable increase in cellular growth in the presence of CXCL12 (100 ng/ml). The results showed that the combination of CXCL12 and CXCR4 could induce a significant increase in cell growth, while SIN repressed the cellular proliferation triggered by combining CXCL12 and CXCR4 to a certain extent (**Figures 1E, F** **)**. These results indicate that SIN suppresses HCC cell growth by regulating the CXCL12-CXCR4 axis.

### CXCL12-CXCR4 Axis Was Involved in the Effect of Sinomenine Hydrochloride on Hepatocellular Carcinoma Cell Apoptosis

**Figure 2:**
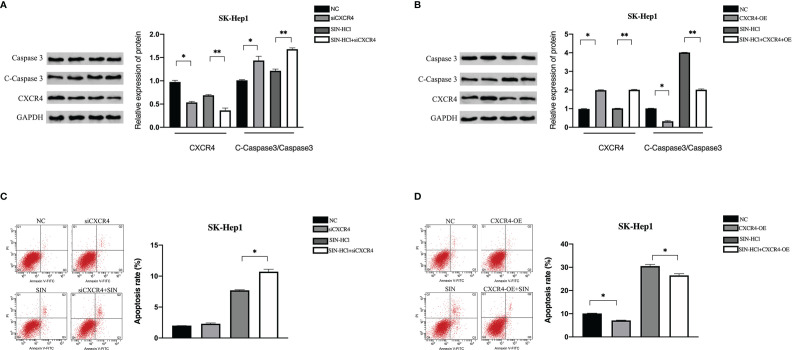
CXCL12-CXCR4 axis was involved in the effect of sinomenine hydrochloride on HCC cell apoptosis. CXCR4 knockdown and overexpression were detected in SK-Hep1 cells. **(A, B)** WB was utilized to identify the expression of apoptotic protein in SK-Hep1 cells. **(C, D)** Programmed cell death was identified *via* flow cell technique. *P < 0.05, **P < 0.01.

Next, we determined whether SIN affected apoptosis of HCC cells through the CXCL12-CXCR4 axis. The expression of apoptosis-related protein Caspase 3 was identified. As unveiled by immunoblotting, the knockout of CXCR4 and sinocine hydrochloride exposure both elevated caspase 3 cleavage in SK-Hep1 cells (**Figure 2A**). Moreover, the highest expression of lysed Caspase 3 was discovered in the sinoline hydrochloride in combination with CXCR4 knockout group in SK-Hep1 cells. By comparison, CXCR4 overexpression decreased sinomenine-triggered cleavage caspase 3 stimulation (**Figure 2B**). The role of CXCR4 modulation in sinomenine-triggered apoptosis in SK-Hep1 cells was studied *via* flow cell technique. SK-Hep1 cells were cultured for 48 hours in the presence of 0.25 mM sinomenine HCl, CXCR4 silenced, or 0.25 mM sinomenine HCl combined with CXCR4 silenced. SK-Hep1 cells were cultured for 48 h under the condition of 0.25-mM SIN and CXCR4 overexpression or 0.25-mM SIN and CXCR4 overexpression. The percentage of apoptosis of SK-Hep1 cells was 8.13 ± 0.45 in SIN group and 10.75 ± 0.29% in SIN in combination with siCXCR4 group, indicating that the knockout of CXCR4 elevated sinomenine-triggered programmed cell death of SK-Hep1 cells (**Figure 2C**). By comparison, the proportion of programmed cell death of SK-Hep1 cells in the SIN group was 30.52 ± 0.74, while the percentage of SK-Hep1 cells in the SIN combined with CXCR4 overexpression group was 26.19% ± 0.28%, indicating that CXCR4 overexpression reduced sinomenine-induced apoptosis of SK-Hep1 cells (**Figure 2D**). Those outcomes suggest that CXCR4 knockout enhances the susceptibility of SK-Hep1 cells to sinoline hydrochloride *via* causing programmed cell death. By comparison, CXCR4 overexpression decreased the susceptibility of HCC cells to sinoline hydrochloride *via* causing programmed cell death.

### CXCL12-CXCR4 Axis Participated in the Effect of Sinomenine Hydrochloride on the Aggression of Hepatocellular Carcinoma Cells

**Figure 3:**
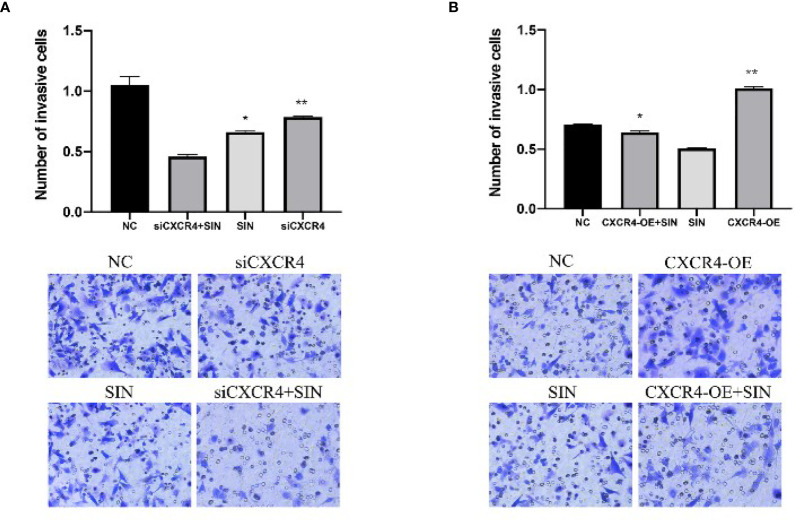
The CXCL12-CXCR4 axis participates in the effect of sinomenine hydrochloride on HCC cell aggression. CXCR4 knockdown and overexpression in SK-Hep1 cells. **(A, B)** Transwell assay for cell invasion (×200). *P < 0.05, **P < 0.01.

To investigate the effects of CXCR4 on sinoline-induced invasion of SK-Hep1 cells, Transwell invasion assay with matrix gel coating was used. In the SIN (0.25 mM) treatment group, the aggression of SK-Hep1 cells was significantly reduced, while CXCR4 knockdown further intensified the invasion of SK-Hep1 cells induced by SIN (**Figure 3A**). In contrast, CXCR4 overexpression reversed the decreased aggression of SK-Hep1 cells induced by sinoline hydrochloride (**Figure 3B**). Therefore, SIN repressed the activity and invasive ability of HCC cells and increased the apoptosis of HCC cells. In conclusion, CXCL12-CXCR4 axis is involved in the role of SIN in HCC cellular viability, invasion, and programmed cell death.

### The CCL21-CCR7 Axis Participated in the Effect of Sinomenine Hydrochloride on Hepatocellular Carcinoma Cells

**Figure 4:**
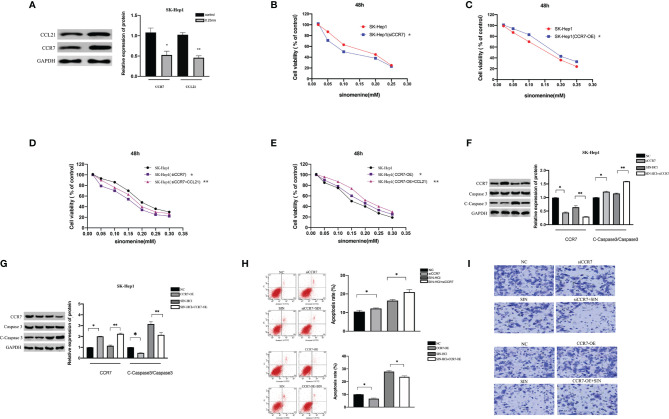
The CCL21-CCR7 axis participates in the effect of sinomenine hydrochloride on HCC cells. **(A)** WB assay of the expression of CCR7 and CCL21 in SK-Hep1 cells. **(B, C)** CCK-8 experiments examine the effect of CCR7 knockout and CXCR7-overexpressing cells on cell proliferation. **(D, E)** CCK-8 experiments on the effect of CCR7-transfected SK-Hep1 cells on cell proliferation in the presence of CCL21 (80 ng/ml). **(F, G)** WB assay of apoptosis-related protein expression in SK-Hep1 cells. **(H)** Apoptosis was detected by flow cytometry. **(I)** Transwell assay for cell invasion (×200). *P < 0.05, **P < 0.01.

CC chemokine ligand 21/chemokine receptor 7 (CCL21/CCR7) facilitates growth and metastases of a variety of cancers (14). This prompted us to explore if SIN also modulates the expression of CCL21/CCR7 in HCC. WB confirmed that CXCR7 participated in the modulation of HCC cells by SIN. As presented by **Figure 4A**, the expressions of CCR7 and CCL21 were remarkably regulated downward in SK-Hep1 cells after SIN (0.25 mM) treatment. To characterize the effects of CCR7 on HCC, our team downregulated or overexpressed CCR7 in SK-Hep1 cells. CCK-8 showed that SK-Hep1 cells with CCR7 knockdown grew more slowly, while CXCR7-overexpressed cells proliferated more rapidly than control cells (**Figures 4B, C** **)**. In addition, SK-Hep1 cells transfected with CCR7 displayed a remarkable increase in cellular growth in the presence of CCL21 (80 ng/ml). The results showed that the binding of CCL21 and CCR7 could induce a significant increase in cell growth, while SIN repressed the cellular proliferation triggered *via* the binding of CCL21 and CCR7 to a certain extent (**Figures 4D, E** **)**. Those outcomes reveal that SIN also regulates the CCL21-CCR7 axis in HCC cells to suppress the growth of oncocytes. Afterward, whether SIN also influences HCC cellular apoptosis through the CCL21-CCR7 axis was determined. WB analysis showed that both CCR7 silencing and sinobine hydrochloride treatment increased caspase 3 cleavage in SK-Hep1 cells (**Figure 4F**). In addition, the highest expression of lysed Caspase 3 was discovered in the sinoline hydrochloride in combination with CCR7 knockout group in SK-Hep1 cells. By comparison, CCR7 overexpression reduces sinomenine hydrochloride-induced activation of cleaved caspase 3 (**Figure 4G**). The effect of CCR7 regulation on sinoline-triggered programmed cell death in SK-Hep1 cells was studied by flow cytometry (**Figure 4H**). SK-Hep1 cells were cultivated for 48 h under the condition of 0.25 mM SIN, CCR7 knockdown, or 0.25 mM SIN combined with CCR7 knockdown. SK-Hep1 cells were overexpressed with SIN and CCR7 at 0.25 mM or SIN combined with CCR7 at 0.25 mM for 48 h. The percentage of apoptosis of SK-Hep1 cells was 7.52% ± 0.42% in SIN group and 8.02% ± 0.37% in SIN in combination with siCXCR4 group, indicating that CCR7 knockout elevated SIN-triggered programmed cell death of SK-Hep1 cells. Apoptosis assay showed that CCR7 overexpression reduced sinoline-triggered programmed cell death of SK-Hep1 cells. By comparison, apoptosis rate was remarkably elevated when CCR7 was silenced in SK-Hep1 cells. Transwell analysis showed that CCR7 knockdown attenuated the invasiveness of SK-Hep1 cells (**Figure 4I**). On the contrary, CCR7 overexpression increased SIN-induced decreased aggression of SK-Hep1 cells. Our results confirm that the CCL21-CCR7 axis is vital for the regulation of sinoline hydrochloride-affected HCC cell proliferation, invasiveness, and apoptosis.

### Tumor-Derived DNA Can Activate CXCL12-CXCR4 and CCL21-CCR7 Axes of Hepatocellular Carcinoma Cells, While Sinoline Hydrochloride Can Inhibit CXCL12-CXCR4 and CCL21-CCR7 Axes of Hepatocellular Carcinoma Cells

**Figure 5:**
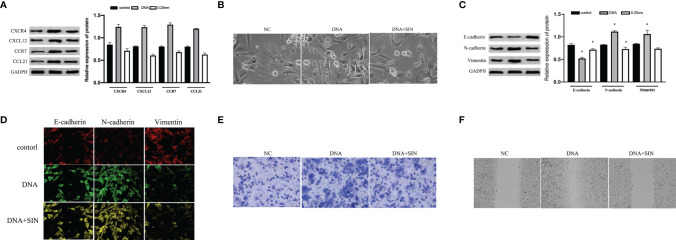
Tumor-derived DNA can activate the CXCL12-CXCR4 and CCL21-CCR7 axes of HCC cells, while sinomenine hydrochloride has the effect of inhibiting the CXCL12-CXCR4 and CCL21-CCR7 axes of HCC cells. **(A)** The protein expression of CXCL12, CXCR4, CCL21, and CCR7 was examined by Western blot (WB) analysis after transfection of tumor-derived DNA. **(B)** Cytomorphological analysis (×200). **(C)** WB assay to examine the expression of E-cadherin, N-cadherin, and vimentin. **(D)** Immunofluorescence for E-cadherin, N-cadherin, and vimentin (×200). **(E)** Transwell analysis of HCC cell invasion (×200). **(F)** Scratch assay to examine HCC cell migration (×200). *P < 0.05.

Cancer-originated DNA was separated from Lewis cell line. Tumor-derived DNA was transfected into SK-Hep1 cells by Lipofectamine 2000. As revealed by WB analyses, the expression of CXCL12, CXCR4, CCL21, and CCR7 proteins in SK-Hep1 cells was significantly increased after transfection with tumor-derived DNA in contrast to the blank control group (**Figure 5A**). The outcomes showed that tumor-derived DNA activated the CXCL12-CXCR4 and CCL21-CCR7 axes in HCC, while sinoline hydrochloride inhibited the CXCl12-CXCR4 and CCL21-CCR7 axes in SK-Hep1 cells. Next, we investigated whether tumor-derived DNA and SIN participated in the modulation of epithelial–mesenchymal transition (EMT) in HCC cells. Cell morphological results showed that tumor-derived DNA increased the mesenchymal morphology of SK-Hep1 cells, and SIN reduced this morphological change (**Figure 5B**). WB was further investigated to determine whether tumor-derived DNA and SIN affected the protein expression levels of E-cadherin, N-cadherin, and vimentin. As shown in **Figure 5C**, tumor-derived DNA significantly downregulated the expression of E-cadherin and upregulated the expression of N-cadherin and vimentin at the protein level. However, sinobine hydrochloride could reverse the effect of tumor-derived DNA on EMT-related markers in SK-Hep1 cells. Representative immunofluorescence was shown in **Figure 5D**, which showed results similar to WB. Transwell analysis showed that tumor-derived DNA remarkably elevated the invasiveness of SK-Hep1 cells, while sinobine hydrochloride reversed the upregulation of tumor-derived DNA on the invasiveness of SK-Hep1 cells (**Figure 5E**). Scratch test results showed that tumor-derived DNA increased HCC cell migration, while sinoline hydrochloride inhibited HCC cell migration *in vitro* (**Figure 5F**). In conclusion, our results indicate that tumor-derived DNA regulates the CXCL12-CXCR4 axis and CCL21-CCR7 axis, which directly affects the metastasis and aggression of HCC cells, while sinoline hydrochloride can reverse the effects of tumor-derived DNA on HCC cells.

### Sinomenine Hydrochloride Interferes With Tumor-Derived DNA and Affects ERK/MMP Signaling Pathway Through CXCL12/CXCR4 Axis in Hepatocellular Carcinoma Cells

**Figure 6:**
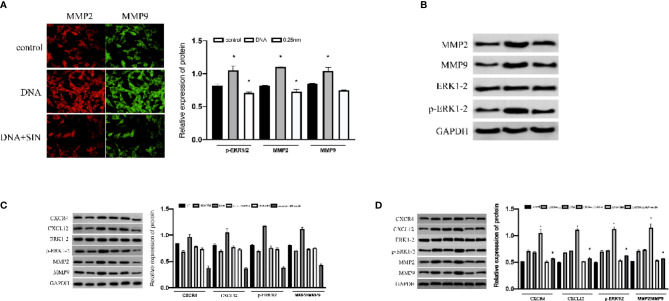
Interference of tumor-derived DNA with sinomenine hydrochloride affects the ERK/MMP signaling pathway through the CXCL12/CXCR4 axis in HCC cells. **(A)** Immunofluorescence examination of the expression of p-ERK1/2 and MMP2/9 in cells (×200). **(B)** WB assay to examine the expression of p-ERK1/2 and MMP2/9 in cells. **(C)** The expression of p-ERK1/2, MMP2, and MMP9 proteins in SK-Hep1 cells after siCXCR4 transfection. **(D)** The expression of p-ERK1/2, MMP2, and MMP9 proteins in SK-Hep1 cells after CXCR4-OE transfection. *P < 0.05.

The effects of mitogen-activated protein kinase (MAPK)/ERK path on tumor has been widely confirmed, which not only participates in oncocyte proliferative activity and angiogenesis but also is vital for cancer aggression and migration (15). Research has revealed that the CXCL12/CXCR4 axis is implicated in a variety of biofunctions of cancers *via* stimulating ERK1/2 (16). For the sake of exploring the effects and causal links of tumor-derived DNA in HCC cell metastasis. We examined the effect of tumor-derived DNA and sinomenine hydrochloride on the phosphorylation of ERK 1/2 in HCC cells and further studied its regulation of MMP expression in HCC cells. The results showed that tumor-derived DNA-treated SK-Hep1 cells exhibited significantly higher P-ErK1/2 and MMP2/9 activity, while sinobine hydrochloride reduced this increase (**Figure 6A**). In addition, tumor-derived DNA remarkably regulated protein expression levels of P-ERK1/2 and MMP2/9 upward, whereas sinobine hydrochloride reduced this upregulation (**Figure 6B**, P < 0.05). Those outcomes revealed that tumor-derived DNA activated ERK/MMP signaling pathway in HCC cells, and sinoline hydrochloride significantly inhibited the ERK/MMP signaling pathway activated by tumor-derived DNA. We further examined whether tumor-derived DNA influenced the ERK/MMP pathway in HCC cells *via* the CXCL12-CXCR4 chemokine axis. As shown in **Figure 6C**, the expressions of P-ErK1/2, MMP2, and MMP9 in SK-Hep1 cells were remarkably reduced after siCXCR4 transfection in contrast to the group without siCXCR4 transfection (DNA group and siCXCR4+DNA group, DNA+SIN group, and siCXCR4+DNA+SIN group were compared respectively). Compared with the untreated sinomenine group, the protein expressions of P-ErK1/2, MMP2, and MMP9 were further inhibited in the sinomenine group (DNA group and DNA+sinomenine group, siCXCR4+DNA group, and siCXCR4+DNA+sinomenine group). We further transfected SK-Hep1 cell lines with CXCR4 plasmid to determine whether tumor-derived DNA and the effect of sinine hydrochloride on HCC cells were related to the CXCL12-CXCR4 chemokine axis. After transfection of tumor-derived DNA, the expression of CXCL12-CXCR4 axis, P-ERK1/2, and MMP2/9 proteins was further significantly increased under CXCR4 overexpression. SIN inhibited this increase to some extent (**Figure 6D**).

### Sinomenine Hydrochloride Interferes With Tumor-Derived DNA and Affects the ERK/MMP Signaling Pathway Through the CCL21-CCR7 Axis in Hepatocellular Carcinoma Cells

**Figure 7:**
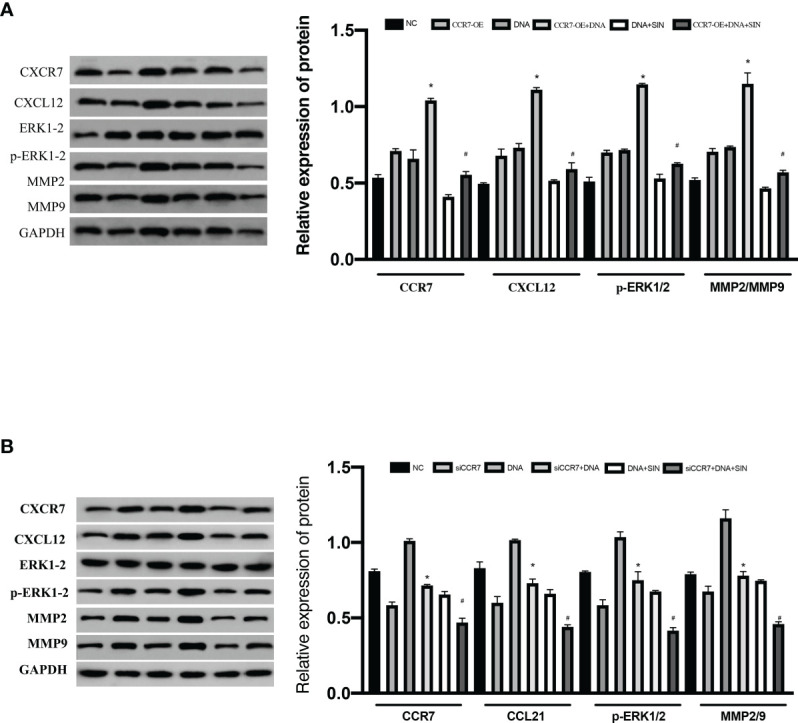
Interfering with tumor-derived DNA by sinomenine hydrochloride affects the ERK/MMP signaling pathway through the CCL21-CCR7 axis in HCC cells. **(A)** The expression of p-ERK1/2, MMP2, and MMP9 proteins in SK-Hep1 cells after siCCR7 transfection. **(B)** The expression of p-ERK1/2, MMP2, and MMP9 proteins in SK-Hep1 cells after CCR7-OE transfection. *P < 0.05(compared with NC), ^#^P<0.05(compared with DNA+SIN group).

The CXCL12/CXCR4 axis has been reported to be involved in a variety of biofunctions of cancers *via* stimulating ERK1/2. Recent reports suggest that CCR7 facilitates cellular proliferative activity *via* the ERK path. WB analyses were completed to explore whether tumor-derived DNA and sinobine hydrochloride modulate ERK/MMP signaling pathway by regulating the CCL21-CCR7 axis in HCC cells. To determine whether the CCL21-CCR7 axis participates in the modulation of tumor-derived DNA and sinobine hydrochloride on ERK1/2 and MMP2/9 signaling pathways, CCR7 was knocked down by siRNA in HCC cells to detect the expression changes of ERK1/2 and MMP2/9. The loss of CCR7 expression leads to the decline of P-ERK1/2 and MMP2/9 (**Figure 7A**), suggesting that CCR7 regulates p-ERK1/2 and MMP2/9 signal paths. The expression of P-ERK1/2, MMP2, and MMP9 protein was further inhibited by sinoline hydrochloride treatment. We also verified the above results from the opposite direction by overexpressing CCR7 in HCC cells (**Figure 7B**). The expression of P-ERK1/2 and MMP2/9 was upregulated after transfection with CCR7 plasmid (**Figure 7B**). Those outcomes reveal that sinobine hydrochloride reverses the role of tumor-derived DNA in HCC cells *via* stimulating the ERK1/2/MMP2/9 signal path regulated by the CCL21-CCR7 axis.

### Tumor-Derived DNA Promoted the Invasion of Hepatocellular Carcinoma Cells Through the CXCL12-CXCR4 Axis and the CCL21-CCR7 Axis, and Sinoline Hydrochloride Had a Suppressive Effect on the Invasion of Hepatocellular Carcinoma Cells

**Figure 8:**
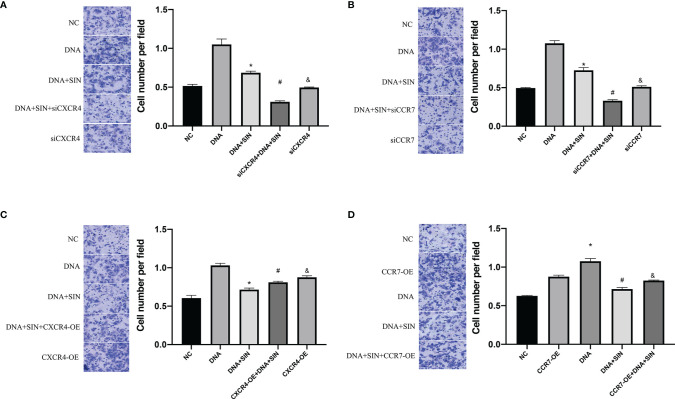
Tumor-derived DNA promotes cell invasion of HCC cells through the CXCL12-CXCR4 axis and CCL21-CCR7 axis, and sinomenine hydrochloride has an anti-invasive effect on HCC cells. **(A, B)** Transwell Matrigel invasion assay of CXCR4 or CCR7 knockdown on cell invasion (×200). **(C, D)** Transwell Matrigel invasion assay on cell invasive ability of CXCR4 or CCR7 overexpression (×200). *P <0.05(compared with NC), #P<0.05(compared with DNA+SIN group), &P<0.05 (compared with siCXCR4+DNA+SIN, or compared with CXCR4-OE+DNA+SIN).

To evaluate the role of tumor-derived DNA in tumor metastasis, Transwell matrix gel invasion was assayed using SK-Hep1 cells with CXCR4 overexpression or silencing. The results showed that tumor-derived DNA promoted SK-Hep1 cell invasion, while CXCR4 knockdown reduced SK-Hep1 cell invasion induced by tumor-derived DNA (**Figure 8A**). Similarly, CCR7 knockdown also reduced SK-Hep1 cell invasion by tumor-derived DNA (**Figure 8B**). SIN significantly suppressed the aggression of SK-Hep1 cells, just as it blocked the CXCL12/CXCR4 axis or CCL21-CCR7 axis. However, the addition of CXCR4 overexpression and CXCL12 further enhanced the increased migration capacity of SK-Hep1 cells induced by tumor-derived DNA (**Figure 8C**). Consistent with the above results, the overexpression of CCR7 and the addition of CCL21 significantly enhanced the invasion ability of SK-Hep1 cells (**Figure 8D**). The addition of SIN repressed CXCR4 overexpression or CCR7 overexpression induced enhanced cell migration. These results further confirmed the previous experimental results that tumor-derived DNA enhanced the invasive ability of SK-Hep1 cells through the CXCL12-CXCR4 axis and the CCL21-CCR7 axis.

### Tumor-Derived DNA Reduced Apoptosis of Hepatocellular Carcinoma Cells by CXCL12-CXCR4 Axis and CCL21-CCR7 Axis, and Sinoline Hydrochloride Reversed the Decrease of Apoptosis

**Figure 9:**
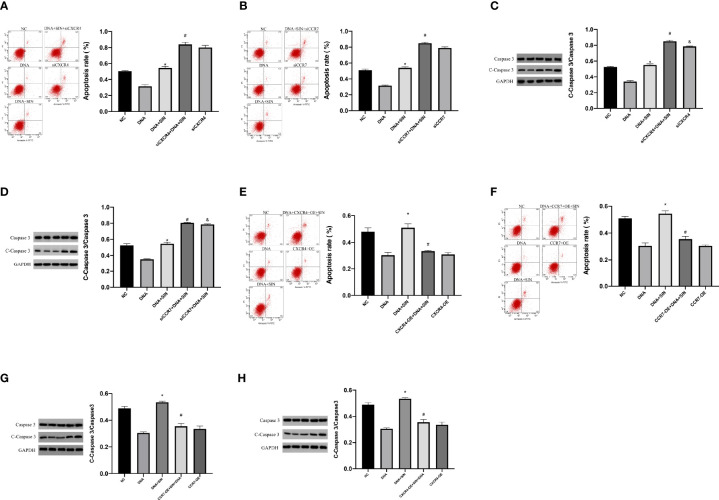
Tumor-derived DNA reduces apoptosis in HCC cells *via* CXCL12-CXCR4 axis and CCL21-CCR7 axis, and sinomenine hydrochloride reverses the reduction in apoptosis. **(A, B)** Flow cytometry to identify the role of siCXCR4 or siCCR7 in the programmed cell death of SK-Hep1 cells in the presence of tumor-derived DNA. **(C, D)** WB assay to identify the expression of apoptotic protein in cells. **(E, F)** The effect of CXCR4 or CCR7 overexpression on the programmed cell death of SK-Hep1 cells in the presence of tumor-derived DNA was detected by flow cytometry. **(G, H)** WB assay to identify the expression of apoptotic protein in cells. *P <0.05(compared with NC), #P<0.05 (compared with DNA+SIN group), &P<0.05 (compared with siCXCR4+DNA+SIN, or compared with CXCR4-OE+DNA7+SIN).

This study subsequently evaluated whether the CXCL12-CXCR4 axis and CCL21-CCR7 axis were involved in the regulation of tumor-derived DNA on apoptosis in HCC cells. Flow cytometry analysis showed that siCXCR4 or siCCR7 increased programmed cell death of SK-Hep1 cells in the presence of tumor-derived DNA (**Figures 9A, B** **)**. Sinoline hydrochloride combined with siCXCR4 or siCCR7 could further increase the programmed cell death of SK-Hep1 cells. In order to reveal in detail that sinobine hydrochloride reversed tumor-derived DNA-induced apoptosis reduction of HCC cells, we examined the protein Caspase 3 levels, and the results were consistent with flow cytometry results, suggesting that siCXCR4 or siCCR7 reversed tumor-derived DNA-induced apoptosis reduction of HCC cells. The addition of SIN further increased HCC cell apoptosis (**Figures 9C, D** **)**. As shown in **Figures 9E** and **F**, apoptosis of SK-Hep1cells was significantly reduced after treatment with tumor-derived DNA combined with CXCR4 or CCR7 overexpression but increased after treatment with sinocine hydrochloride. WB analyses were completed to identify the level of Caspase 3, a protein associated with apoptosis, and the results were consistent with flow cytometry results, indicating that CXCR4 or CCR7 overexpression reduced tumor-derived DNA-induced apoptosis of HCC cells, while sinocine hydrochloride increased Caspase 3 levels (**Figures 9G, H**). In conclusion, these results further support the previous experimental results that tumor-derived DNA reduces the apoptosis of HCC cells through the CXCL12-CXCR4 and CCL21-CCR7 axes, and the addition of sinobine hydrochloride can reverse this reduction.

## Discussion

HCC is a challenging and lethal malignancy with increasing incidence (17). Exogenous or improperly positioned endogenous DNA can stimulate immune responses, and the molecular mechanisms of DNA recognition have received increasing attention. Tumor-derived DNA is fragmented DNA of tumor-derived DNA found in human blood and other biological fluids (18, 19). It carries the complete genome information of tumor-specific genetic changes, which can be detected by detecting the genetic changes associated with the tumor. In recent years, increasing evidence has shown that tumor DNA plays a key role in the pathogenesis of various tumors (20). The mechanism by which DNA enters the cytoplasm of Dendritic Cells (DCs) under physiological conditions *in vivo* is one of the unresolved questions in our current work. Here, our team intends to study and focus on the mechanism by which tumor-derived DNA acts as a carcinogen of liver cancer to promote cancer progression *in vivo* (21, 22). Chemokine signaling has long been involved in cancer progression and metastases through autocrine or paracrine mechanisms. Importantly, in previous studies, the chemokine receptor CXCR4 was found to be overexpressed in HCC samples and HCC cells and was found to enhance HCC growth and invasiveness (17, 23). CXCR4 overexpression may be attracted to specific organs by its ligand CXCL12, which together lead to organ-specific metastasis. The CXCL12-CXCR4 axis promotes tumor growth and angiogenesis and enhances invasion and migration. Our preliminary results indicate that CXCR4 and CXCL12 are highly expressed in HCC cells. Although the CXCL12-CXCR4 axis is critical for HCC, it is involved in different cellular pathways under tissue heterogeneity and different cellular contexts. To date, the biological function of tumor-derived DNA on the CXCL12-CXCR4 axis in HCC has not been reported. Here, we report a novel finding that tumor-derived DNA upregulates the CXCL12-CXCR4 axis in HCC, activates the ERK/MMP signaling pathway, and promotes the proliferation and metastasis of HCC cells.

SIN is an alkaloid monomer extracted from the dried stems of Sinomenine or Sinomenine, is the hydrochloride form of sinomenine. It has a wide range of pharmacological activities and has significant anti-inflammatory, anti-immunity, and antitumor effects. Recently, with the advancement of SIN research, certain new pharmacological effects and mechanisms of sinomenine have been discovered, especially its antitumor effects have been paid more and more attention by scholars at home and abroad. Here we show that SIN can inhibit the upregulation of CXCL12-CXCR4 axis induced by tumor-derived DNA in HCC cells. This research offers enlightenment pertaining to the modulation of CXCL12-CXCR4 axis and HCC cell proliferation and metastasis by SIN. First, the effects of several doses of SIN on HCC cell survival/proliferative activity were evaluated. The outcomes unveiled that SIN exerted suppressive effects on the survival/proliferation of SK-Hep1 cells, and 0.25-mm SIN had the maximum inhibitory effect. Apoptosis is also a major determinant of cell survival/proliferation. Lysed Caspase 3 is considered to be a key executor of apoptosis and can induce apoptosis.

Herein, our team discovered that sinobine hydrochloride elevated the protein level of proapoptotic activator caspase 3, and increased apoptosis was also observed by drain cytology, suggesting that the effect of proapoptotic activator sinobine hydrochloride was related to the regulation of apoptosis-related regulatory factors. CXCL12-CXCR4 axis is overexpressed in HCC cells, and downregulation of CXCL12-CXCR4 axis is beneficial to inhibit cancer growth. The outcomes herein unraveled that SIN reduced the protein levels of CXCL12, CXCR4, CCL21, and CCR7 in HCC cells. Our team discovered that cancer-originated DNA can activate the CXCL12-CXCR4 and CCL21-CCR7 axes in HCC cells, while sinoline treatment reduced the expressions of CXCL12, CXCR4, CCL21, and CCR7 in HCC cells. The MAPK pathway is a prominent mechanism for coordinating transcription factors that are activated by ERK kinase to modulate downstream MMP-2 and MMP-9 genetic expression and afterward elevate cancer onset and development. The aggression and migration of cancer are complicated processes with many factors and steps. MMP-mediated decomposition of exocellular matrixes and basement membranes is a pivotal step in cancer aggression and migration. It has been reported that oral sinobine hydrochloride significantly blocks the stimulation of ERK1/2 in the spinal cord (24). In addition, SIN can effectively reduce the metastases of mankind glioblastoma *via* inhibiting the MMP2/9 signaling pathway (25). Our team discovered that cancer-originated DNA can promote the stimulation of ERK1/2 and MMP2/9 in HCC cells, while cotreatment with sinobine hydrochloride reduces the expression of ERK1/2 and MMP2/9. Brand et al. (26) showed that chemokine CXCL12 can induce colon cancer cells to secrete MMP9 through its specific receptor CXCR4. CXCR4 cellular mutations activate Akt and ERK signaling and promote varying degrees of drug resistance to ibrutinib, bendamustine, fludarabine, bortezomib, and edralicib (27). CXCL12 can also enhance MMP9 secretion of non-Hodgkin's lymphoma (NHL) cells and promote extracellular matrix degradation, thus participating in cell invasion (28).

To further study the mechanism of CXCL12-CXCR4 induced by tumor-derived DNA promoting the aggression of HCC cells *in vitro*. Herein, the effects of CXCR4 knockdown and overexpression on the expression of ERK1/2 and MMP2/9 were also observed. It was found that CXCR4 knockdown downregulated the level of ERK1/2 in SK-Hep1 cells, enhanced the expression and secretion of MMP2/9, and degraded exocellular matrixes and basement membranes, thus promoting the invasion and migration of HCC. In addition, the proapoptotic protein Caspase 3 and apoptosis rate were also significantly downregulated in HCC cells after CXCR4 knockdown and sinoline hydrochloride treatment, suggesting that CXCR4 knockdown also promoted HCC cell apoptosis. CXCR4 knockdown and sinobine hydrochloride cotreatment also further decreased the expression of ERK1/2 and MMP2/9, further repressed the proliferative and migratory activities of SK-Hep1 cells, and further facilitated the programmed cell death of HCC cells. On the contrary, CXCR4 overexpression increased the tumor-derived DNA expression of ERK1/2 and MMP2/9, increased the proliferative and migratory activities of SK-Hep1 cells, and reduced apoptosis. Therefore, regulation of ERK1/2/MMP2/9 signaling pathway may be one of the mechanisms of CXCL12-CXCR4 axis or CCL21-CCR7C axis inhibition and sinoline hydrochloride synergistic anti-HCC. On the contrary, CXCR4 overexpression led to further decreased expression of ERK1/2 and MMP2/9, which also proved that CXCL12-CXCR4 axis or CCL21-CCR7C axis inhibition and sinoline hydrochloride had a synergistic anti-HCC effect.

## Conclusion

These results suggest that inhibition of tumor-derived DNA-induced activation of CXCL12-CXCR4 or CCL21-CCR7C axis has a synergistic anti-HCC effect with sinoline hydrochloride.

## Data Availability Statement

The original contributions presented in the study are included in the article/Supplementary Material. Further inquiries can be directed to the corresponding author.

## Author Contributions

We contributed equally for this work. All authors contributed to the article and approved the submitted version.

## Funding

Clinical Research Plan of SHDC (No.SHDC2020CR2021B).

## Conflict of Interest

The authors declare that the research was conducted in the absence of any commercial or financial relationships that could be construed as a potential conflict of interest.

## Publisher’s Note

All claims expressed in this article are solely those of the authors and do not necessarily represent those of their affiliated organizations, or those of the publisher, the editors and the reviewers. Any product that may be evaluated in this article, or claim that may be made by its manufacturer, is not guaranteed or endorsed by the publisher.
